# Sociodemographic and geographical variation in prescribing psychotropic drugs to children and young people with common mental disorders and Attention Deficit Hyperactive Disorders in North West London: population-based study

**DOI:** 10.1136/bmjopen-2024-094149

**Published:** 2025-11-24

**Authors:** Antonio Ivan Lazzarino, Stephen Russell Naulls, Rinad Bakhti, Steven Hope, Dasha Nicholls, Michaela Otis, Tamsin Robinson, Shamini Gnani, Dougal S Hargreaves

**Affiliations:** 1 School of Public Health, Imperial College London, London, UK; 2 Department of Clinical Neuroscience, Brighton and Sussex Medical School, Brighton, UK; 3 Department of Brain Sciences, Imperial College London, London, UK; 4 NIHR Applied Research Collaboration (ARC) North West London, London, UK; 5 NHS North West London Integrated Care Board, London, UK

**Keywords:** Electronic Health Records, Prescriptions, MENTAL HEALTH, Child & adolescent psychiatry, Health Equity, Attention Deficit Disorder with Hyperactivity

## Abstract

**Abstract:**

**Objectives:**

To estimate the sociodemographic and geographical variation in prescribing selective serotonin reuptake inhibitors (SSRIs) and medications for attention-deficit/hyperactivity disorder (ADHD) to children and young people (CYP) in North West London, UK.

**Design:**

Cross-sectional population-based study.

**Setting:**

General practices in North West London, UK, with data for the period 2020–2022 obtained from the Discover Now platform, which covers approximately 95% of the local population.

**Participants:**

762 390 CYP aged 5–24 years in the year 2022.

**Primary and secondary outcome measures:**

Primary outcome: Prescription rates of SSRIs and ADHD medications. Secondary outcomes: Associations between prescription rates and sociodemographic factors, including age, gender, geographical area (local authority), ethnicity and socioeconomic deprivation (measured using the Index of Multiple Deprivation).

**Results:**

The total sample comprised 762 390 CYP. 2.20% of the sample were prescribed an SSRI (95% CI 2.17% to 2.24%) and 0.50% an ADHD medication (95% CI 0.49% to 0.52%) in years 2020–2022. High deprivation was associated with the highest rates of an SSRI prescription (2.5%). In contrast, low deprivation was associated with the highest rates of an ADHD medication prescription (0.70%). This divergent pattern was evident in some London boroughs and not in others. The relationship between level of area deprivation and prescription rates also differed by borough. Overall, the sociodemographic factors could not explain most of the variation in prescription rates (Pseudo R^2^ 0.18 for SSRI and 0.06 for an ADHD medication).

**Conclusions:**

Prescriptions for common mental disorders and ADHD for CYP from North West London varied by sociodemographic characteristics and London borough of residence, potentially exacerbating mental health inequalities. To monitor and address these inequalities, more extensive use of linked electronic health records should be undertaken; for example, data on mental health diagnosis and service utilisation are needed to investigate the relationship between diagnosis and treatment over time.

**STRENGTHS AND LIMITATIONS OF THIS STUDY**

- The study uses high-quality, population-level health service data (the Discover Now platform), which provides comprehensive coverage for the North West London region, including primary care prescription data, precise geographical information and a range of sociodemographic factors, including the Index of Multiple Deprivation, age, gender and ethnicity.
- The large sample size and wide population coverage improve the generalisability of the results and provide a strong foundation for identifying disparities and informing targeted interventions to reduce health inequalities.
- The Discover Now platform does not include prescribing within the independent private sector, so there is the potential for under-recording of prescription rates, especially in wealthier areas.
- The platform does not capture mental healthcare activity from outside the mental health provider trusts’ remit. This comprises counselling services (including school counselling), talking services and/or other community mental health support services, for example within schools.

## Background

 The escalating global prevalence of mental disorders among children and young people (CYP) has contributed to challenges in accessing appropriate mental healthcare services.^1^ This trend has had notable implications for health providers. For example, the financial burden of drug treatments for depression alone among individuals under 25 years old in England amounted to over £12 million in the fiscal year 2022/2023. Meanwhile, the sharp increase in diagnosis of attention-deficit/hyperactivity disorder (ADHD) and accompanying prescription of medication has led to significant financial costs^1^ and supply shortages, yet the dynamics driving these increases remain unclear.^2^

The patient journey for CYP with mental disorders has multiple stages, from the expression of symptoms and diagnosis, through to the initiation of a treatment regime, and each stage may be influenced by the sociodemographic and cultural circumstances of the CYP.^3^ However, little is known at a population level about how treatment, particularly prescribing practices, for mental disorders varies by these circumstances. Primary care serves as a gatekeeping function in the United Kingdom, with general practitioners (GPs) making referrals to specialist Child and Adolescent Mental Health Services (CAMHS) or, in some areas, paediatric-led neurodevelopmental services. Specialists usually undertake diagnosis and initiate medication, after which GPs are responsible for routine monitoring and repeat prescriptions under shared care arrangements.^4^ Long median waiting times from primary care referral to consulting with a CAMHS specialist remain a significant concern,^5^ with the potential to exacerbate mental health problems and health inequalities in access to care.

Leveraging the Discover Now platform, which integrates comprehensive health data for the population of North West London, provides a unique opportunity to analyse variations in treatment access and utilisation. Using these data, this study explores prescription patterns for prevalent mental health disorders such as depression, anxiety and ADHD among CYP, according to sociodemographic characteristics, with a particular focus on geographical variation, based on local and national National Health Service (NHS) priorities. Insights from these analyses aim to inform the development of [targeted interventions and policy measures to mitigate inequities and better align mental healthcare delivery with need in the future.

## Methods

### Study design

Cross-sectional population survey using linked electronic health records.

### Setting

The North West London Integrated Care System has attempted to collect all health-related information from all residents in that area of the UK within a database called Whole Systems Integrated Care (WSIC). The main objective of the database is to improve the quality of care by making individual-level health information readily available to health professionals.^6^

This study is based on the analysis of deidentified records, which are made available to researchers on request through a research platform called Discover Now.^7^ Discover Now gathers individual-level electronic health records from primary, secondary and tertiary care for all people registered with participating GP practices in North West London, covering approximately 95% of the resident population of 2.4 million people. Discover Now has been in operation since 2015 and gathers information from 365 GP practices, 10 acute and specialist hospitals, 2 mental health trusts, 2 community health trusts and social care providers.^7^ The data source tables have been described elsewhere.^2^ Researchers can link the source tables together using deidentified NHS numbers. Data source refreshes are scheduled every 1–3 months depending on the data source.

### Participants

North West London residents, who were aged 5–24 years in the year 2022.

### Eligibility criteria

All CYP registered at a general practice in North West London were included. We excluded CYP registered with the ‘GP at Hand’ practice in Hammersmith & Fulham as it has registered patients outside North West London (providing digital consultations via a phone app).

### Outcome measure

Our primary outcome measure was the presence of at least one GP prescription for either a common mental disorder (CMD) (depression and/or anxiety disorder) or an ADHD medication in the years 2020–2022. We analysed selective serotonin reuptake inhibitors (SSRIs) and ADHD medications separately. The names of medicines included in the analyses are listed in online supplemental materials appendix 1. We focused on these medicines because: (1) they tend to be specific to mental health diagnoses; (2) they are well coded and (3) they are prescribed frequently enough to examine variation by borough and deprivation index, which would not apply to medications more rarely used in these populations such as antipsychotics and mood stabilisers.

### Exposure variables

The Discover Now Table Patient Index includes sociodemographic information including gender, borough of residence, ethnicity and socioeconomic deprivation. North West London has eight boroughs or Local Authorities: Ealing, Brent, Hillingdon, Hounslow, Harrow, Hammersmith and Fulham, Westminster and Kensington and Chelsea. Ethnicity takes five values (variable named Ethnic Category): Asian, black, mixed, other and white. Ethnicity was unspecified for 5.2% of the study sample, and we included those people in the analysis, considering them as a sixth category (unspecified ethnicity).

For socioeconomic deprivation, the variable named imdrank indicates the English Index of Multiple Deprivation (IMD) for year 2019, which is an area-based index of poverty produced by the UK Ministry of Housing, Communities and Local Government, which is calculated as a weighted score based on seven domains: income, employment, education, health, crime, barriers to housing and services, and living environment.^8^ A higher score indicates lower socioeconomic deprivation. In this study, the score was divided into quintiles, IMD 1–5 with IMD 1 representing the most socioeconomically deprived group.

### Statistical analysis

We calculated crude prescription rates (prevalence) with 95% CIs for the whole sample and after stratification by sociodemographic characteristics including age, gender, ethnicity, borough of residence and IMD quintile. For borough and ethnicity, we used the most populated category as the reference category. This happened to be Ealing for the borough of residence, and white for ethnicity. Adjusted stratum-specific prescription rates were then calculated using logistic regression and indirectly standardising to the observed distribution of covariates for the whole sample using the *margins* command in Stata V.15.

## Results

We identified 920 104 people who were aged 5–24 years in 2022. We excluded 154 117 people who were not resident in one of the eight boroughs of North West London and a further 3519 people who were registered with the GP practice called GP at Hand which registers patients from outside North West London. We also excluded nine people with unspecified primary care network, and a further 69 people with a missing value for gender. The final analytical sample comprised 762 390 CYP aged between 5 and 24 years.

### Descriptive statistics

The prevalence of a prescription for an SSRI was 2.20% (95% CI 2.17% to 2.24%), while the prevalence of a prescription for medication to treat ADHD was 0.50% (95% CI 0.49% to 0.52%).

All sociodemographic variables were associated with medication rates, with marked variation in the rate of prescribing of SSRIs and ADHD medication by age, gender, ethnicity, socioeconomic deprivation and borough of residence. For SSRIs, higher rates of prescription were observed for those in the oldest age group (18–24 years), females, of white ethnicity, residents in the most deprived areas (quintile 1), and those living in the borough of Hammersmith and Fulham (table 1A). For ADHD medication, higher prescription rates were observed for those aged 13–17 years, males, of white ethnicity, residents in the least deprived areas (quintile 5), and those living in the Boroughs of Hammersmith and Fulham or Kensington and Chelsea (table 1B).

**Table 1:** (A) Sample description of discover Now/WSIC participants aged 5–24 years in 2022 by SSRI prescription status and (B) sample description of discover Now/WSIC participants aged 5–24 years in 2022 by ADHD medicine prescription status

(A) Factor and category		SSRI prescribed in years 2020–2022		P value
		No	Yes	
		N=745 587	N=16 803	
Age in years		14.8 (5.9)	21.3 (2.4)	<0.001
Age category	5–12	293 262 (100.0%)	72 (0.0%)	<0.001
	13–17	174 158 (99.4%)	1125 (0.6%)	
	18–24	278 167 (94.7%)	15 606 (5.3%)	
Gender	Male	381 612 (98.7%)	5216 (1.3%)	<0.001
	Female	363 975 (96.9%)	11 587 (3.1%)	
Ethnicity	White	273 497 (96.8%)	9041 (3.2%)	<0.001
	Asian	209 219 (98.7%)	2672 (1.3%)	
	Other	110 667 (98.0%)	2306 (2.0%)	
	Black	71 855 (98.1%)	1422 (1.9%)	
	Unspecified	39 328 (99.2%)	320 (0.8%)	
	Mixed	41 021 (97.5%)	1042 (2.5%)	
Socioec. deprivation	1 (most deprived)	147 413 (97.5%)	3826 (2.5%)	<0.001
(quintile of IMD)	2	149 534 (97.9%)	3176 (2.1%)	
	3	154 595 (97.8%)	3404 (2.2%)	
	4	148 552 (97.9%)	3153 (2.1%)	
	5 (least deprived)	145 493 (97.8%)	3244 (2.2%)	
London Borough	Ealing	128 188 (97.8%)	2825 (2.2%)	<0.001
	Brent	127 409 (98.3%)	2187 (1.7%)	
	Hillingdon	118 654 (97.8%)	2731 (2.2%)	
	Hounslow	98 339 (97.9%)	2155 (2.1%)	
	Harrow	97 210 (98.3%)	1698 (1.7%)	
	Hammersmith and Fulham	62 947 (96.5%)	2280 (3.5%)	
	Westminster	62 379 (97.5%)	1587 (2.5%)	
	Kensington and Chelsea	50 461 (97.4%)	1340 (2.6%)	

(B) Factor and category		ADHD medicines prescribed in years 2020–2022		P value
		No	Yes	
		N=758 569	N=3821	
Age in years		15.0 (5.9)	16.9 (4.4)	<0.001
Age category	5–12	292 644 (99.8%)	690 (0.2%)	<0.001
	13–17	173 944 (99.2%)	1339 (0.8%)	
	18–24	291 981 (99.4%)	1792 (0.6%)	
Gender	Male	384 208 (99.3%)	2620 (0.7%)	<0.001
	Female	374 361 (99.7%)	1201 (0.3%)	
Ethnicity	White	280 160 (99.2%)	2378 (0.8%)	<0.001
	Asian	211 504 (99.8%)	387 (0.2%)	
	Other	112 521 (99.6%)	452 (0.4%)	
	Black	73 027 (99.7%)	250 (0.3%)	
	Unspecified	39 600 (99.9%)	48 (0.1%)	
	Mixed	41 757 (99.3%)	306 (0.7%)	
Deprivation	1 (most deprived)	150 543 (99.5%)	696 (0.5%)	<0.001
(quintile of IMD)	2	152 124 (99.6%)	586 (0.4%)	
	3	157 309 (99.6%)	690 (0.4%)	
	4	150 908 (99.5%)	797 (0.5%)	
	5 (least deprived)	147 685 (99.3%)	1052 (0.7%)	
London Borough	Ealing	130 625 (99.7%)	388 (0.3%)	<0.001
	Brent	129 117 (99.6%)	479 (0.4%)	
	Hillingdon	120 936 (99.6%)	449 (0.4%)	
	Hounslow	99 961 (99.5%)	533 (0.5%)	
	Harrow	98 288 (99.4%)	620 (0.6%)	
	Hammersmith and Fulham	64 706 (99.2%)	521 (0.8%)	
	Westminster	63 526 (99.3%)	440 (0.7%)	
	Kensington and Chelsea	51 410 (99.2%)	391 (0.8%)	

Data are means (SD) for continuous variables and N (row percentage) for categorical variables. P values are from independent t-tests for continuous variables and from χ² test for categorical variables.

ADHD, attention-deficit/hyperactivity disorder; SSRI, selective serotonin reuptake inhibitor; WSIC, Whole Systems Integrated Care.

Rates of prescription by age differed by gender. For SSRIs, prescription rates were low for males and females until adolescence, with marked increases for both sexes, but notably for females, after 16 years (online supplemental materials figure 1A). For ADHD medication, there was a gradual increase in prescription rates for females until 18 years when they stabilised, while for males there was a steeper increase in the prescription rate for males until 18 years, followed by a marked decline (online supplemental materials figure 1B).

### Multivariable regression

Table 2A,B shows odds of being prescribed an SSRI or ADHD medication, respectively, according to sociodemographic characteristics.

**Table 2:** (A) Output from a multiple logistic regression model showing mutually adjusted ORs for the prescription of an SSRI (OR), with 95% CIs and p values and (B) output from a multiple logistic regression model showing mutually adjusted ORs for the prescription of medicine for ADHD (OR), with 95% CIs and p values

(A) Factor	Category	ORs	(95% CIs)	P value
London Borough	Ealing	1	(reference category)	
	Brent	0.74	(0.70 to 0.79)	<0.001
	Hillingdon	1.13	(1.07 to 1.20)	<0.001
	Hounslow	1.20	(1.13 to 1.27)	<0.001
	Harrow	0.95	(0.90 to 1.02)	0.149
	Hammersmith and Fulham	1.05	(0.99 to 1.11)	0.105
	Westminster	0.80	(0.75 to 0.85)	<0.001
	Kensington and Chelsea	0.84	(0.79 to 0.90)	<0.001
Socioeconomic deprivation	1 (most deprived)	1	(reference category)	
(quintile of IMD)	2	0.81	(0.77 to 0.85)	<0.001
	3	0.82	(0.78 to 0.86)	<0.001
	4	0.77	(0.74 to 0.81)	<0.001
	5 (least deprived)	0.75	(0.71 to 0.79)	<0.001
Gender	Female	2.17	(2.10 to 2.24)	<0.001
Ethnicity	White	1	(Reference category)	
	Asian	0.38	(0.36 to 0.39)	<0.001
	Other	0.57	(0.54 to 0.59)	<0.001
	Black	0.60	(0.57 to 0.64)	<0.001
	Unspecified	0.21	(0.19 to 0.23)	<0.001
	Mixed	0.93	(0.87 to 1.00)	0.041
Age	1-year increase	1.32	(1.31 to 1.33)	<0.001

(B) Factor	Category	ORs	(95% CIs)	P value
London Borough	Ealing	1	(Reference category)	
	Brent	1.35	(1.18 to 1.55)	<0.001
	Hillingdon	1.18	(1.03 to 1.35)	0.018
	Hounslow	1.98	(1.74 to 2.26)	<0.001
	Harrow	2.18	(1.92 to 2.49)	<0.001
	Hammersmith and Fulham	2.02	(1.77 to 2.30)	<0.001
	Westminster	1.81	(1.57 to 2.08)	<0.001
	Kensington and Chelsea	1.90	(1.65 to 2.19)	<0.001
Socioeconomic deprivation	1 (most deprived)	1	(Reference category)	
(Quintile of IMD)	2	0.93	(0.83 to 1.04)	0.19
	3	0.94	(0.84 to 1.04)	0.247
	4	1.05	(0.95 to 1.17)	0.331
	5 (least deprived)	1.24	(1.12 to 1.37)	<0.001
Gender	Female	0.44	(0.41 to 0.48)	<0.001
Ethnicity	White	1	(Reference category)	
	Asian	0.22	(0.20 to 0.25)	<0.001
	Other	0.45	(0.41 to 0.50)	<0.001
	Black	0.44	(0.38 to 0.50)	<0.001
	Unspecified	0.13	(0.10 to 0.18)	<0.001
	Mixed	0.93	(0.82 to 1.05)	0.218
Age	1-year increase	1.06	(1.06 to 1.07)	<0.001

ADHD, Attention Deficit Hyperactivity Disorder; IMD, Index of Multiple Deprivation; SSRI, selective serotonin reuptake inhibitor.

All covariates were associated with the outcomes after the mutual adjustment, with patterns similar to those reported in the descriptive results above, except for Borough of residence. The models explained only a small amount of variation in prescription rates (Pseudo R^2^ 0.18 for SSRI and 0.06 for ADHD medication). Standardised rates for each covariate after adjustment for all other covariates, derived from these logistic regression models are shown for SSRI (online supplemental materials figures 2A and 3A) and ADHD medication (online supplemental materials figures 2B and 3B).

### Borough of residence

There were clear differences in the distributions of all sociodemographic variables for each borough of residence (online supplemental materials table 1), indicating diversity in the CYP populations of these areas. Patterns of prescribing by borough were not consistent between SSRIs and ADHD medicines in the adjusted models (table 2A,B and online supplemental materials figure 4A,B). For SSRIs, results were mixed. Compared with Ealing (baseline), two boroughs had higher prescription rates, three boroughs had lower prescription rates, while rates in the remaining boroughs were no different to Ealing. In contrast, for ADHD, all boroughs had higher rates of prescription compared with Ealing.

**Figure 1:**
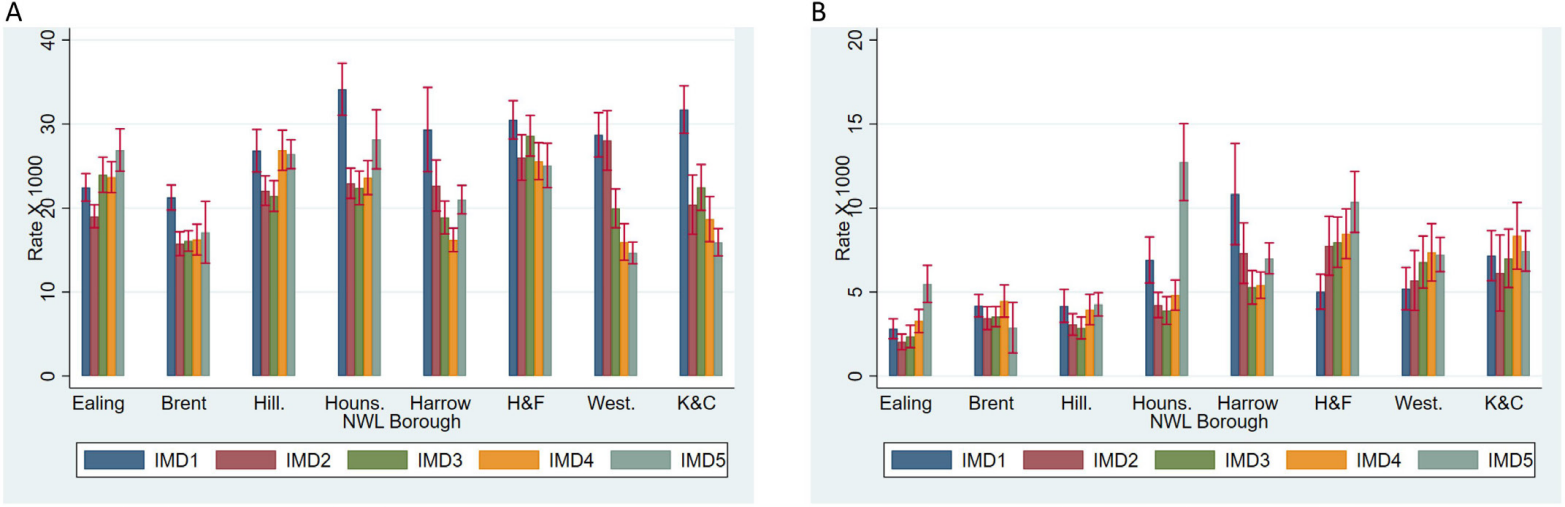
(**A**) SSRI prescription rates, stratified by North West London (NWL) borough and socioeconomic deprivation quintile (95% CIs). (**B**) ADHD medicine prescription rates, stratified by NWL borough and socioeconomic deprivation quintile (95% CIs). IMD, Index of Multiple Deprivation; ADHD, Attention Deficit Hyperactivity Disorder; SSRI, selective serotonin reuptake inhibitor.

There was considerable variation in prescription rates after stratification by borough and socioeconomic deprivation, adjusting for the remaining sociodemographic characteristics. For SSRIs, prescription rates were highest for those living in the most deprived areas (IMD1), except for Ealing and Hillingdon (figure 1A). For ADHD medication, the results were more variable according to borough, with generally higher rates of prescription for those residents in the least deprived areas (IMD5), although this pattern appeared stronger in some boroughs (eg, Hounslow, Ealing and Hammersmith and Fulham) and was weaker or not apparent in other boroughs (figure 1B).

## Discussion

We found wide variation in prescribing rates for SSRIs and ADHD medication by age, ethnicity, socioeconomic deprivation and borough in North West London. Nevertheless, despite the inclusion of a wide range of sociodemographic characteristics likely to influence prescribing, only a small proportion of the prescribing variation could be explained, suggesting the influence of additional unidentified factors.

Rates of SSRI prescriptions were found to be higher among CYP who live in the most deprived neighbourhoods. This finding is consistent with evidence elsewhere and may reflect underlying differences in the prevalence of CMDs such as depression and anxiety; higher prevalence of CMD is associated with greater deprivation and is mostly treated with SSRI medication.^9 10^ Research suggests that CYP with depression and anxiety living in deprived areas are more likely to be offered drug treatment than talking therapies, an equally effective treatment.^11^,^13^ Further, individuals may receive and/or require SSRI treatment when they are sicker because they present later because of many factors, including reduced access to services, higher levels of stigma and/or lower levels of mental health literacy.^14^

Demand for timely access to publicly funded mental health services is also greater in more deprived areas due to overall system resource constraints with less capacity to manage the negative impacts of treatment delays or fragmented services.^5^ Services in wealthier areas may be better integrated and comprehensive, leading to more appropriate prescribing of SSRI prescriptions. In contrast, when there are fragmented services in deprived areas, care may be disjointed, treatment delayed and SSRI prescribing is inconsistent.^15^ This can result in perpetuating inequities.

We observed an association between higher SSRI prescription rates and increasing age that reflects wider age trends in prevalence of CMD and differences in prescribing guidelines between age groups. We found variation in SSRI prescribing rates by gender and ethnicity, which may reflect differences in prevalence and expression of CMDs among groups. Cultural and linguistic factors can influence the recognition, expression and treatment of mental health symptoms among CYP and their families.^16^ Ethnic differences in accessing mental healthcare have been reported in the nationally representative Mental Health of Children and Young People in England Survey, with children from ethnic minorities less likely to contact any professional services.^17^ Evidence from a scoping review of qualitative studies also demonstrates ethnic difference in care seeking and access to mental healthcare that arise from a range of factors, including lack of information and trust in professionals, and social stigma and cultural expectations.^18^

Research also indicates that biases and stereotypes inherent in mental healthcare provision may influence clinical prescribing habits^19 20^ and a lack of culturally competent services may contribute to under/over diagnosis and treatment among minority ethnic young people. This may widen treatment gaps and disparities, including SSRI prescription rates. Complex referral pathways can create hurdles that affect individuals differently based on socioeconomic status, gender and ethnicity; those from wealthier backgrounds may navigate these pathways more effectively, leading to quicker access to mental health assessments and treatment such as medication. This may exacerbate existing disparities between those who have the resources and knowledge to navigate intricate referral processes, with those who do not; for example, self-referral services for talking therapies for CMDs.

We found differences in ADHD prescription rates between those aged <18 years and those aged 18–24 years. This may be partly explained by the role that UK GPs play as gatekeepers for accessing ADHD treatment.^21^ Previous research conducted among GPs found low levels of confidence in initiating psychotropic medication for ADHD treatment without specialist input and variation in adherence to National Institute for Health and Care Excellence (NICE) ADHD guidelines, especially among younger children.^1922^,^24^ Our findings may reflect a growing confidence among GPs to start stimulant medication in the adult population^23 25^ between specialists and GPs, especially on the transition from children to adult mental health services. The transition between children’s and adult services across both primary and secondary care has also been a major focus of attention for improvement across a range of mental health conditions,^26^,^28^ as many CYP experience poor continuity of care.

There has also been a broadening of the phenotype for ADHD in diagnostic manuals in recent decades, which likely contributes to higher diagnosis and therefore higher prescription rates.^29^ NHS England data from 2022/2023 shows that 150 233 people aged under 25 years received stimulant prescriptions for treatment of ADHD, totalling approximately £51 m per year.^1^ Indeed, this is the first time within the period covering these statistics that adults have received more prescriptions of this type than children. This likely reflects both an ageing population of CYP entering adulthood with a diagnosis of ADHD and better recognition and diagnosis of ADHD in the young adult population.^30^

Our study found higher rates of ADHD prescriptions in the least deprived areas, potentially due to reduced stigma and timely access to diagnosis and drug treatment.^31^ Parents and carers are important in supporting access to mental healthcare; parents with higher levels of education and from wealthier backgrounds may be more familiar with the condition of ADHD, more effective in advocating for their child’s needs and navigating the healthcare system,^32^ with a greater likelihood of diagnosis and prescribing of ADHD treatment. Additionally, schools in affluent areas may offer better mental health support for students with ADHD, facilitating earlier diagnosis and drug treatment. There may be better access to specialised ADHD services, including child psychiatrists and specialist paediatricians in less deprived areas. Clinical and practitioner experience suggests that many ADHD diagnoses occur outside the funded health system (NHS), which may lead to an underestimation of ADHD prescriptions in least deprived populations.^30^

Cross-national data have shown consistent trends of rising SSRI and ADHD medication prescriptions among CYP, although with considerable variation in prevalence between countries that is likely to reflect factors including differences in diagnostic criteria, health systems, regulations, treatment guidelines and cultural attitudes.^33^ These temporal trends have also been observed in the UK, continuing from prepandemic through to the postpandemic period. Variations in prescriptions have been shown at regional and local level in the UK for SSRI and ADHD medication,^34^ with explanations including the potential impact of inequities in care and service provision.^35^ In North West London, variations in prescribing for SSRI and ADHD medications were found between boroughs of residence after accounting for other sociodemographic factors. Possible explanations for these borough differences in prescribing include historical factors about service provision, such as the number of child psychiatrists in post, dedicated care pathways for emotional or neurodevelopmental disorders and possible differences in services provided by the two Mental Health Trusts operating in North West London. Further investigation is required to identify specific factors that drive these differences between boroughs.

### Study strengths and limitations

A key strength of the study lies in its use of a high-quality, population-level dataset from the Discover Now platform, which provides comprehensive and accurate coverage of the population of the North West London region. This dataset includes robust coding of prescription data, ensuring reliable identification of SSRIs and ADHD medication usage. Additionally, it offers precise geographical information, allowing for analysis of regional variation in prescribing patterns. The inclusion of the IMD and other sociodemographic factors, such as age, gender and ethnicity, further enhances the study’s capacity to explore and quantify the relationship between socioeconomic factors and prescription trends, making the findings relevant for public health planning. The large sample size and wide population coverage improves the generalisability of the results and provides a strong foundation for identifying disparities and informing targeted interventions to reduce health inequalities.

However, the study also has limitations. Most of the variation in prescription rates in this study was not explained by the sociodemographic characteristics available, suggesting the presence of other influences, including individual factors (eg, diagnosis and severity), sociorelational factors (eg, family and peer relations), and health-related economic factors (eg, non-drug public spending and the prescribing attitudes of clinicians). IMD is an area-based measure and so is an imperfect measure of the socioeconomic circumstances of the child. We were unable to investigate clinical diagnosis, referral processes or waiting times for mental health (CAMHS) services. This prevented our ability to analyse the patient journey from primary care to CAMHS and identify potential barriers to accessing services. The Discover Now research platform excludes data on prescribing from independent private healthcare providers, so there is likely an overall under-recording of prescription rates. This is likely to lead to an underestimation of prescription rates in wealthier areas, where private healthcare use is higher. Additionally, the platform does not capture mental health care activity occurring outside the mental health provider trusts’ remit. This comprises counselling services (including school counselling), talking therapies and other community mental health support services. Given that NICE recommends talking therapies, such as cognitive–behavioural therapy for depression and anxiety as the preferred treatment approach in CYP, it is important to note that our study’s focus on prescription data may not fully capture the prevalence of a condition or the use of treatments. A future study could use diagnostic data. At the time of these analyses, the WSIC dataset had limited CAMHS data. However, the dataset is expanding as more data are being included.

The study focused on the period 2020–2023 and excludes data before the COVID-19 pandemic. The pandemic increased levels of poor mental health and disorders among CYP. This has continued in subsequent years, and it is likely that analysis of prescribing patterns postpandemic reflects contemporary prescribing patterns. However, it is important to continue to monitor for variations and changes in prescribing patterns.

### Conclusions and implications for health policy and further research

Prescribing patterns reflect a combination of inter-linking factors that can be characterised at an individual and system level and include population sociodemographic characteristics, individual prescribing preferences of physicians, clinical thresholds for referral pathways, and availability and access to specialist mental health services. Further research should be carried out to explore other factors that may be implicated in these results, and the interplay between multiple factors. However, these descriptive findings have implications for public health commissioners and service level planning by highlighting variations in prescribing that may inform design, evaluation and targeting of local and national strategies to improve outcomes and reduce inequalities in young people’s mental health.

## Supplementary material

10.1136/bmjopen-2024-094149 online supplemental file 1

10.1136/bmjopen-2024-094149 online supplemental file 2

## Data Availability

Data may be obtained from a third party and are not publicly available.
