# Supplementary material for: Sociodemographic and geographical variation in prescribing psychotropic drugs to children and young people with common mental disorders and Attention Deficit Hyperactive Disorders in North West London: population-based study
Source: BMJ Open. 2025 Nov 24;15(11):e094149. doi: 10.1136/bmjopen-2024-094149 (PMC12645602; doi:10.1136/bmjopen-2024-094149)

**Supplementary material**

|  | | **North West London Borough** | | | | | | |
| --- | --- | --- | --- | --- | --- | --- | --- | --- |
|  | Ealing | Brent | Hill. | Houns. | Harrow | H & F | Westm. | K&C |
| **Factor and**  **category** | N=131,013 | N=129,596 | N=121,385 | N=100,494 | N=98,908 | N=65,227 | N=63,966 | N=51,801 |
| Age in years* | 14.9 (5.9) | 15.0 (5.9) | 14.7 (5.9) | 14.4 (5.8) | 14.6 (5.8) | 15.7 (6.2) | 15.9 (6.1) | 15.7 (6.1) |
| Age category* | | | | | | | | |
| 5-12 | 50,963 (38.9%) | 49,360 (38.1%) | 48,328 (39.8%) | 42,306 (42.1%) | 39,863 (40.3%) | 23,204 (35.6%) | 21,384 (33.4%) | 17,926 (34.6%) |
| 13-17 | 30,226 (23.1%) | 30,928 (23.9%) | 29,066 (23.9%) | 24,612 (24.5%) | 24,632 (24.9%) | 13,058 (20.0%) | 12,096 (18.9%) | 10,665 (20.6%) |
| 18-24 | 49,824 (38.0%) | 49,308 (38.0%) | 43,991 (36.2%) | 33,576 (33.4%) | 34,413 (34.8%) | 28,965 (44.4%) | 30,486 (47.7%) | 23,210 (44.8%) |
| Gender* | | | | | | | | |
| Male | 67,138 (51.2%) | 65,704 (50.7%) | 63,361 (52.2%) | 51,838 (51.6%) | 51,407 (52.0%) | 30,932 (47.4%) | 31,281 (48.9%) | 25,167 (48.6%) |
| Female | 63,875 (48.8%) | 63,892 (49.3%) | 58,024 (47.8%) | 48,656 (48.4%) | 47,501 (48.0%) | 34,295 (52.6%) | 32,685 (51.1%) | 26,634 (51.4%) |
| Ethnicity* | | | | | | | | |
| White | 45,068 (34.4%) | 38,569 (29.8%) | 45,805 (37.7%) | 33,068 (32.9%) | 30,520 (30.9%) | 36,034 (55.2%) | 27,492 (43.0%) | 25,982 (50.2%) |
| Asian | 40,855 (31.2%) | 37,921 (29.3%) | 41,337 (34.1%) | 33,999 (33.8%) | 40,247 (40.7%) | 4,696 (7.2%) | 8,781 (13.7%) | 4,055 (7.8%) |
| Other | 18,738 (14.3%) | 21,840 (16.9%) | 12,522 (10.3%) | 10,379 (10.3%) | 12,314 (12.4%) | 10,009 (15.3%) | 15,959 (24.9%) | 11,212 (21.6%) |
| Black | 13,582 (10.4%) | 19,322 (14.9%) | 10,899 (9.0%) | 7,127 (7.1%) | 7,344 (7.4%) | 7,261 (11.1%) | 4,188 (6.5%) | 3,554 (6.9%) |
| Unspecified | 5,410 (4.1%) | 5,331 (4.1%) | 4,725 (3.9%) | 11,336 (11.3%) | 3,718 (3.8%) | 2,604 (4.0%) | 3,191 (5.0%) | 3,333 (6.4%) |
| Mixed | 7,360 (5.6%) | 6,613 (5.1%) | 6,097 (5.0%) | 4,585 (4.6%) | 4,765 (4.8%) | 4,623 (7.1%) | 4,355 (6.8%) | 3,665 (7.1%) |
| Socioeconomic deprivation (quintile of IMD)* | | | | | | | | |
| 1 (most deprived) | 32,182 (24.6%) | 39,484 (30.5%) | 16,709 (13.8%) | 14,934 (14.9%) | 4,720 (4.8%) | 18,085 (27.7%) | 12,636 (19.8%) | 12,489 (24.1%) |
| 2 | 34,733 (26.5%) | 29,400 (22.7%) | 29,252 (24.1%) | 30,067 (29.9%) | 8,715 (8.8%) | 9,110 (14.0%) | 7,029 (11.0%) | 4,404 (8.5%) |
| 3 | 20,659 (15.8%) | 37,353 (28.8%) | 25,272 (20.8%) | 22,469 (22.4%) | 20,959 (21.2%) | 12,991 (19.9%) | 9,982 (15.6%) | 8,314 (16.0%) |
| 4 | 26,277 (20.1%) | 18,503 (14.3%) | 18,188 (15.0%) | 23,857 (23.7%) | 34,096 (34.5%) | 13,802 (21.2%) | 9,264 (14.5%) | 7,718 (14.9%) |
| 5 (least deprived) | 17,162 (13.1%) | 4,856 (3.7%) | 31,964 (26.3%) | 9,167 (9.1%) | 30,418 (30.8%) | 11,239 (17.2%) | 25,055 (39.2%) | 18,876 (36.4%) |
|  | | | | | | | | |

**Table 1. Sample description of Discover Now/WSIC participants aged 5–24 years in 2022.**

Data are means (S.D.) for continuous variables and N (column percentage) for categorical variables. * p<0.001. P values are from ANOVA for continuous variables and from Chi-squared test for categorical variables. H&F, Hammersmith & Fulham; Hill, Hillingdon; Houns, Hounslow; K&C, Kensington & Chelsea; West, Westminster


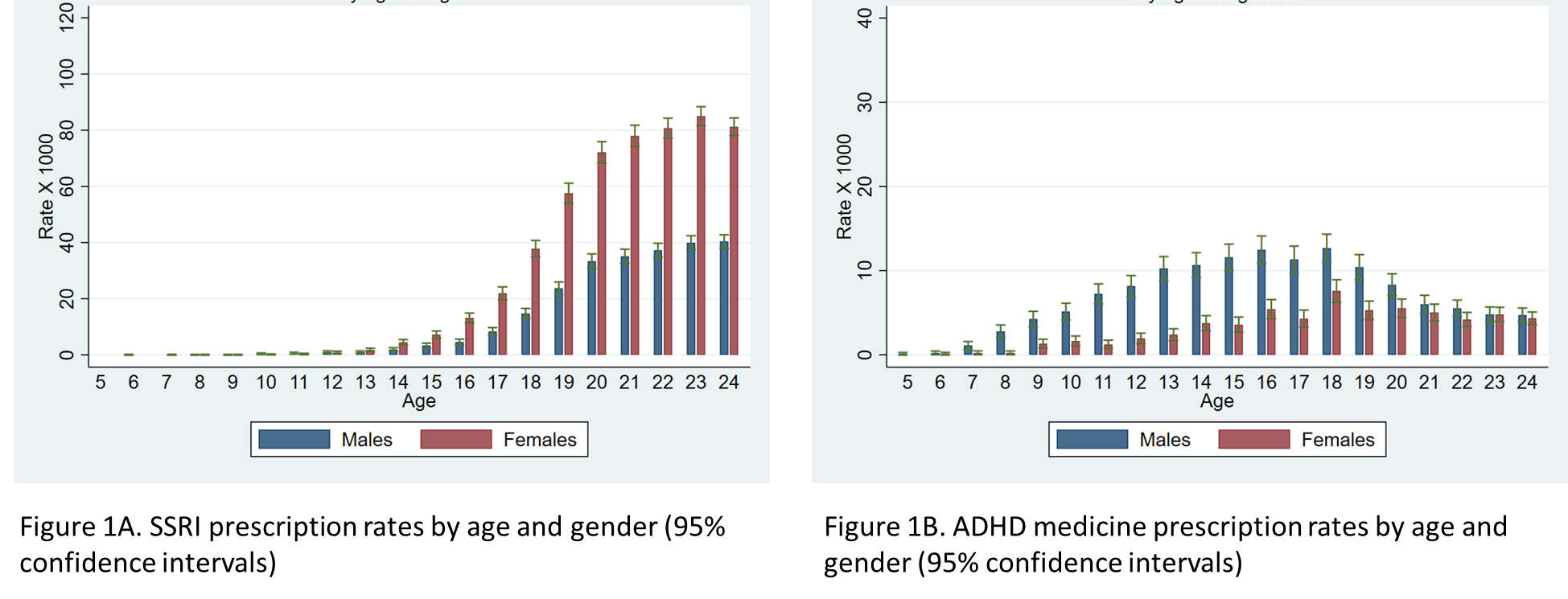


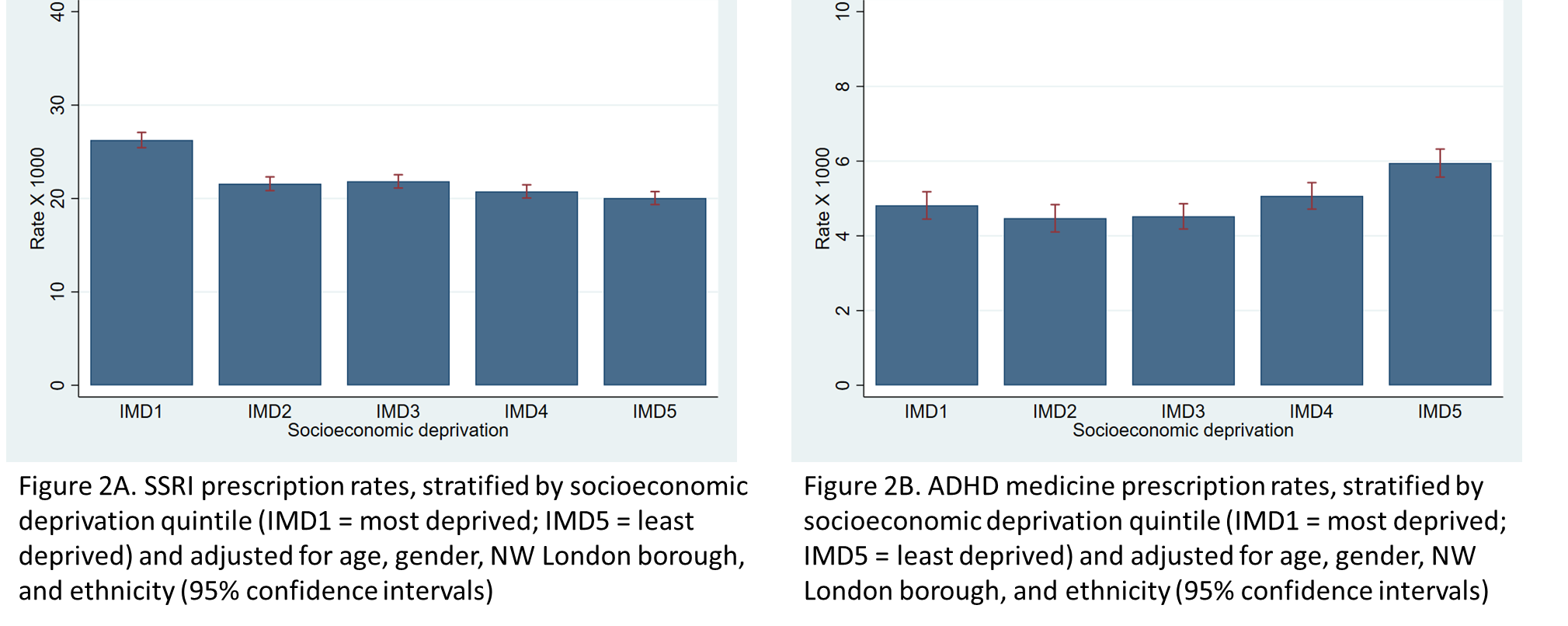


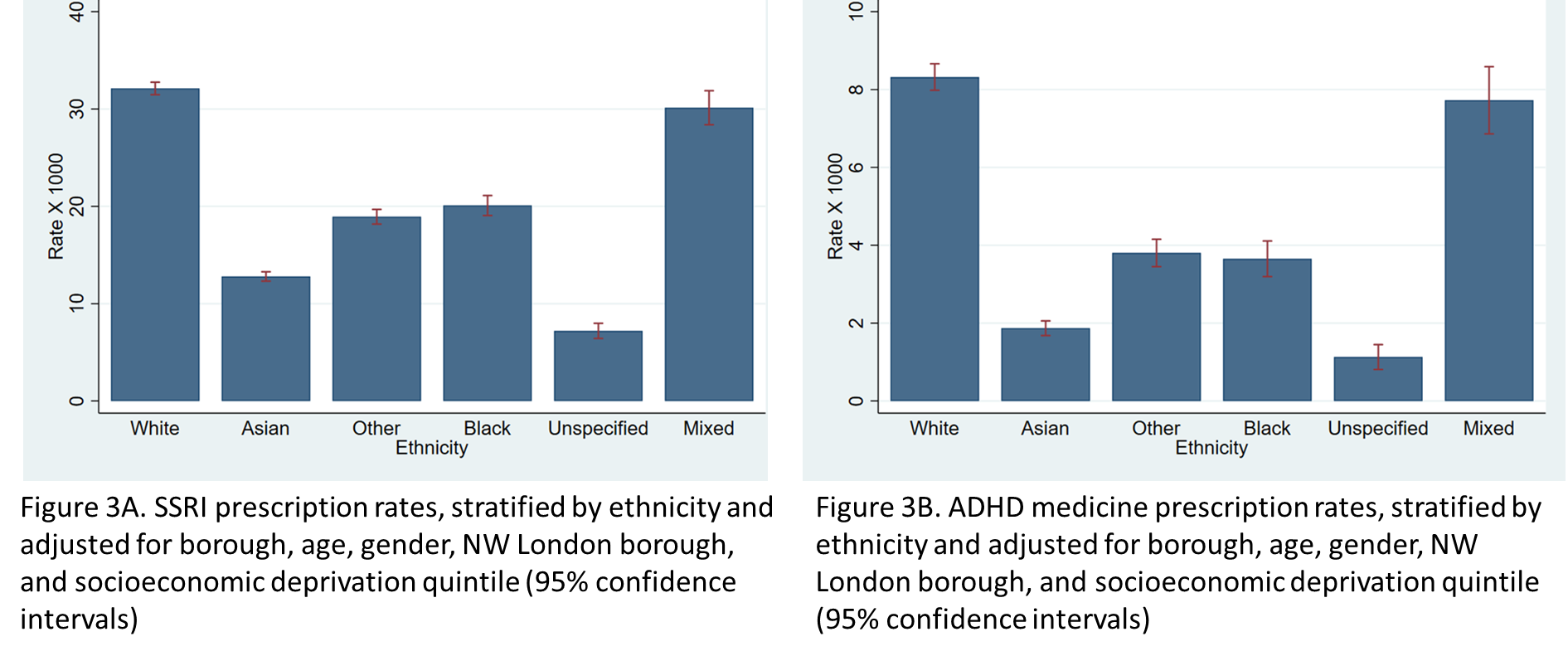


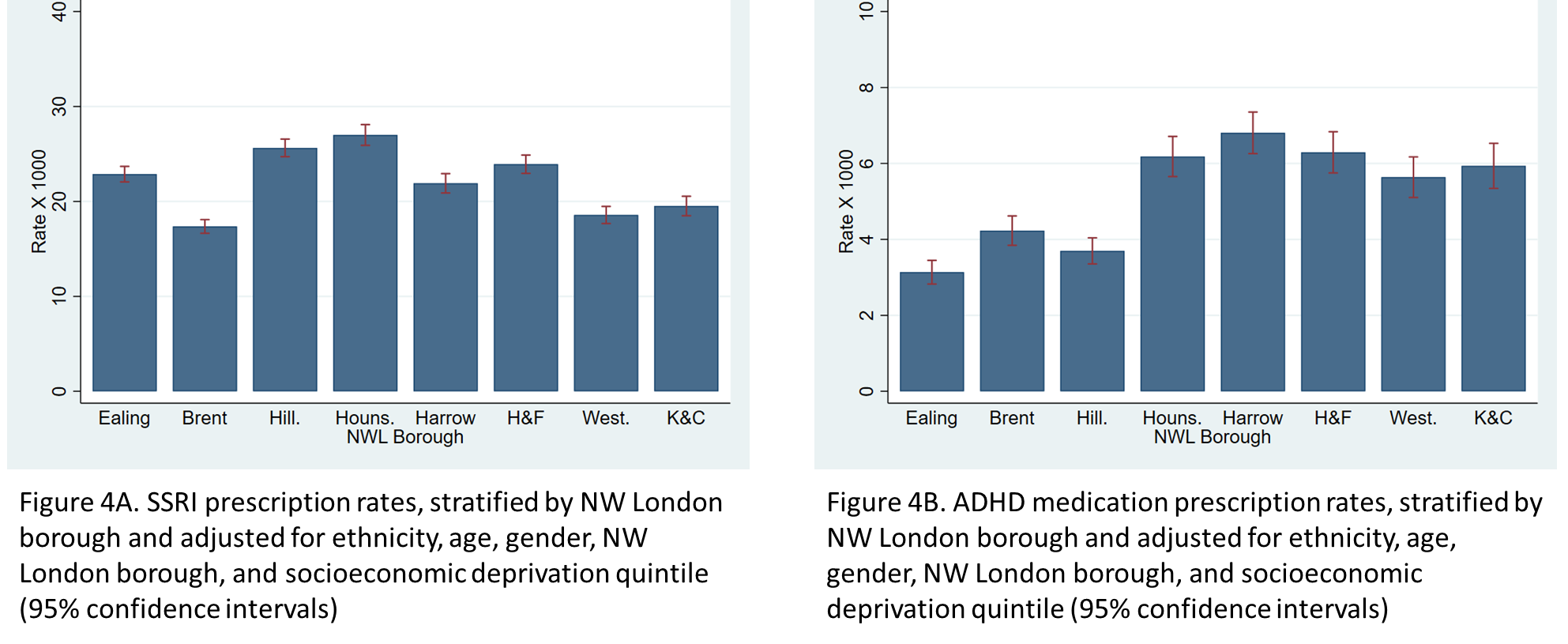

Supplement: online supplemental file 1 [file bmjopen-15-11-s001.docx]
