# Supplementary material for: Sociodemographic and geographical variation in prescribing psychotropic drugs to children and young people with common mental disorders and Attention Deficit Hyperactive Disorders in North West London: population-based study
Source: BMJ Open. 2025 Nov 24;15(11):e094149. doi: 10.1136/bmjopen-2024-094149 (PMC12645602; doi:10.1136/bmjopen-2024-094149)
Supplement: online supplemental file 2 [file bmjopen-15-11-s002.docx]

**Appendix 1**

List of drugs included in the analysis.

| **Name** | **Type** |
| --- | --- |
| BRINTELLIX | SSRI |
| CIPRALEX | SSRI |
| CIPRAMIL | SSRI |
| CITALOPRAM | SSRI |
| CONCERTA | ADHD |
| DAPOXETINE | SSRI |
| DELMOSART | ADHD |
| EQUASYM | ADHD |
| ESCITALOPRAM | SSRI |
| FAVERIN | SSRI |
| FLUOXETINE | SSRI |
| FLUVOXAMINE | SSRI |
| LUSTRAL | SSRI |
| MEDIKINET | ADHD |
| METHYLPHENIDATE | ADHD |
| OXACTIN | SSRI |
| PAROXETINE | SSRI |
| PRILIGY | SSRI |
| PROZAC | SSRI |
| RITALIN | ADHD |
| SEROXAT | SSRI |
| SERTRALINE | SSRI |
| VORTIOXETINE | SSRI |
