# Supplementary material for: Sociodemographic and geographical variation in prescribing psychotropic drugs to children and young people with common mental disorders and Attention Deficit Hyperactive Disorders in North West London: population-based study
Source: BMJ Open. 2025 Nov 24;15(11):e094149. doi: 10.1136/bmjopen-2024-094149 (PMC12645602; doi:10.1136/bmjopen-2024-094149)
Supplement: Supplementary file 3 [file bmjopen-2024-094149.reviewer_comments.pdf]

PEER REVIEW HISTORY

BMJ Open publishes all reviews undertaken for accepted manuscripts. Reviewers are asked to complete a checklist review form and are provided with free text boxes to elaborate on their assessment. These free text comments are reproduced below.

ARTICLE DETAILS

Title (Provisional)

Sociodemographic and geographical variation in prescribing psychotropic drugs to children and young people with common mental disorders and Attention Deficit Hyperactive Disorders in Northwest London: population-based study.

Authors

Lazzarino, Antonio Ivan; Naulls, Stephen Russell; Bakhti, Rinad; Hope, Steven; Nicholls, Dasha; Otis, Michaela; Robinson, Tamsin; Gnani, Shamini; Hargreaves, Dougal S

VERSION 1 - REVIEW

**Reviewer**	**1**
**Name**	**Amendola, Simone**
**Affiliation**	**Independent researcher**
**Date**	**14-Nov-2024**
**COI**	**None**

Dear Editor and Dear Authors,

I appreciate the invitation and opportunity to review the manuscript "*Sociodemographic and geographical variation in prescribing psychotropic drugs to children and young people with common mental disorders and Attention Deficit Hyperactive Disorders in Northwest London: population-based study*". The present analysis benefited from the use of a comprehensive coverage of SSRI and ADHD medications prescribed in the Northwest London region. It is indeed an interesting paper exploring associations between prescribing patterns and geographical indicators. According to my opinion, the clarity and rigor of the manuscript need to be further improved as well as the reporting in the results section and the discussion of some of the results. The use of a consistent structure when presenting the results and when discussing them (e.g. starting with presenting/discussing findings related to age first, then gender, the borough of residence, ethnicity, and lastly socioeconomic deprivation) may be of help in organizing the text and improving the reading flow. Below are my comments and suggestions. I would gladly review the second version of this manuscript. Best regards

Title and abstract

1. Reading the abstract, it is evident that the analysis deals with SSRI and ADHD medications.

I believe it would be better to specify this already in the title rather than using "*psychotropic drugs for … disorders*". Or did the authors also analyze indications for prescribing those drugs (please note that this is not reported in the abstract)?

2. The time during which prescriptions were analyzed could be added to the abstract under "*setting*". 2020-2022 is reported among the results but it is unclear whether this was the period of interest for this study.
3. Results are only partially reported and focus on deprivation in the current form. Preference needs to be given to results from logistic regression and divergent findings according to each drug group.
4. Abstract conclusions: are you sure that they match your results? For example, it is stated that "*Mental health care … does not appear to be well matched to need and may be exacerbating inequalities*.". However, did you analyze which type of intervention was suggested/provided besides drugs or CYP needs? This is not clear in the abstract. Furthermore, this is a cross-sectional study, and the data analyzed could not inform on cause-effect relationship (i.e. exacerbating inequalities). Under results, it was also reported that "*sociodemographic factors could not explain most of the variation in prescription rates*". Finally, what is intended with *"More extensive use of linked electronic health records"* may be also briefly clarified (e.g. with examples between brackets) in the abstract and addressed in the discussion section.
5. May more specific analysis be needed to inform on the relationship between prescribing and socioeconomic deprivation, also considering the severity of mental health symptoms for which drugs were prescribed and other therapeutic interventions?

Background

6. The topic of pharmacological interventions for mental health difficulties is introduced on line 14. However, more contextual data is needed to provide a better picture for readers who are not from the UK. For example, are mental health difficulties of CYP primarily treated with drugs rather than other types of treatments? This section would benefit from a discussion of available evidence on preferred/recommended mental health treatments for CYP in the UK and London specifically, if any.
7. It seems that this analysis was entirely exploratory despite focusing on sociodemographic factors (i.e. no specific hypothesis to be tested). This could be clarified at the end of the background section.

Methods

8. The reason to exclusively analyze SSRI and ADHD drugs among many other drugs available on the market needs to be clarified as well. Is this perhaps a limitation of the study that aimed to focus on psychotropic drugs for common mental disorders?
9. Similarly, the reason to focus on the period 2020-2022 and not on the years before (from 2015 to 2020) needs some explanation. Such a choice might have also made possible the analysis of disparities in prescribing trends.
10. Could the focus on residents only represent a bias in this analysis? More than 150 thousand CYP were excluded. Did the authors test differences between included and excluded participants in the variables considered in this study? Any difference in prescription prevalence and sociodemographic indicators?
11. It would be good to add excluded drugs within each category, if any, to Appendix 1.

Results

12. The 95% CI for prevalence could be added to the column "yes" (i.e. prescription status) in Tables 1A and 1B.
13. Table 2 is difficult to read in its current form. I would suggest excluding raw numbers to only report prevalence.
14. To facilitate results comparison between bivariate association (table 1a and 1b) and multivariate association (table 3a and 3b) logistic regression models should be preferred to test bivariate associations (using the same reference category as in the multivariate models) and odds ratio could be added to table 1a and 1b rather than Chi-square test. The chi-square test alone says nothing about where the difference in the distribution occurred.

    If the chi-square test is preferred standardized residuals should be reported and their effect interpreted.
15. Considering that it is common to observe significant p-values with large samples, how did the authors discriminate whether their results were also (from a public health or clinical perspective) meaningful? Previous recommendations about OR effect size interpretation might help here. This would also guide the authors to focus on more important, key, or meaningful findings in their discussion. The following may be of help https://doi.org/10.1080/03610911003650383.
16. I would encourage the authors to differentiate three sections of the results, a first one describing results from bivariate associations, a second one on those from multivariate analysis, and a third one describing the figures (subheading might be useful here).
17. Regarding the description of the figures, a clearer description could be that: in Figure 5a, the lower prescription of SSRI for all less deprived groups (compared to the most deprived, IMD1) is evident overall, except for Ealing and Hill. In Figure 5b there is more variability related to the findings from the logistic regression of ADHD drug prescription higher for the least deprived group (IMD5) compared to the reference (IMD1) (and no difference between IMD1 and other groups). Figure 5b suggests that the significant result is possibly primarily explained by prescriptions in Houns and HeF with Harrow representing an exception (where we see that the effect is reversed). However, all of this is a description and was not formally tested in a subgroup/moderation analysis. I believe that if the authors intend to focus on the discussion of these specific results they need to perform additional analysis. Lacking such an analysis, I recommend deleting the description from these results from the abstract.

Discussion

18. Lines 11-12: There seems to be no linear relationship according to your findings but only that "the MOST deprived group" showed higher SSRI prescription compared to other less deprived and non-deprived groups. Please change accordingly.
19. Lines 52: The authors found an effect of ethnicity even after controlling socioeconomic deprivation and geographic area of NWL and ascribed this effect to the possible occurrence of bias, stereotypes, and competency within the services. Is this difference between ethnicities observed in access to non-drug mental health interventions as well?
20. Page 7 on the bottom: "*We found differences in ADHD prescription rates between those aged <18 years and those aged 18-24 years*". I cannot find this analysis in the text. If you refer to the data displayed in Figure 1b, then the pattern is a bit more complex. There "seems" to be (because no test was conducted, maybe I am missing something here) a gradual linear increase in ADHD drug prescription in females while male prescription rates peaked at 18 years old and then decreased. These findings may merit further discussion.

21. Line 25: "*we found higher rates of ADHD prescriptions in less deprived areas*". The difference was significant only between the least and the most deprived areas. The most deprived area did not differ from areas with "intermediate" deprivation. Please change accordingly.
22. Could the significant difference be because hyperactivity and inattention in the least deprived area may be considered socially inadmissible needing correction, something "out of order"? When this is not the case for the least deprived and intermediates areas? The authors could consider this additional explanation.
23. A relevant part of the results section is focused on prescriptions according to London Borough. However, a discussion of the findings is completely lacking.
24. The discussion could be organized in subheadings, e.g. with one paragraph on social deprivation and another on geographical variation.
25. I also encourage the authors to assume a more critical perspective and include a paragraph discussing the limitations of this study.
26. The section about study implications primarily relies on disparities in waiting times but this aspect was not analyzed in the present study. Please base the study implications on the study findings.
27. Conclusions need restructuring despite the fact that the first part (lines 17-22) is well integrated with the rest of the text. The authors could capitalize on that to highlight how their study informs or advances current knowledge, i.e. one or a few take-home messages resulting from the findings of the study.

**Reviewer**	**2**
**Name**	**Ward, John**
**Affiliation**	**University of Exeter**
**Date**	**19-Jan-2025**
**COI**	**None**

Thank you for giving me the opportunity to review this interesting manuscript. I think that the subject matter, variations in prescribing considering social factors, is very important. I am impressed by the size of the dataset used in this study.

My main comment would be that the analysis is very interesting, but feels that the background/discussion and conclusion have been tacked on without a good narrative thread in the paper. A unifying thread through the paper would help make for a more robust argument.

As a general comment, I think that Northwest should be two words (North West), but I will leave this as a journal house style matter.

Specific comments;

Title: you are missing an "a" from "a population based study"

Abstract:

Your results section needs tidying up; it reads as cumbersome and there are issues with the grammar.

Also, you say ADHD medication and ADHD drug; please be consistent.

Background:

I think that your background is far too short, and doesn't justify why you go onto the look at the question that you do.

I agree that CYP mental health is multi-faceted and prescribing is expensive, but I want to know a bit more about why this justifies your study and how your study would help you address the problems you have identified. You sort of do this in the last paragraph of the background but it's quite vague and does not pin onto any existing literature. As another general note, you comment a couple of times own the costs of prescribing but for unclear reason (both in the background and in the discussion).

Methods:

The description of the dataset is good; thank you.

Re the participants; you say that they have to be in the dataset in 2022, but the sample period is between 2020-22. Did they have to be in the dataset for this entire period, or just 2022?

Was any de-duplication of the dataset required (I'm assuming not if by NHS number).

Was this study pre-registered (not that it had to be, but would be good to know for the reader).

There is no justification as to why using prescription rates in this study? It would be good to know why specifically you have chosen prescribing patterns, and why specifically SSRIs and ADHD medications.

Was the IMD rank for the GP practices or for the patients themselves?

Results:

I would not use the word correlation unless you have calculated a correlation.

"More deprivation in certain ones"—too informal.

Discussion:

Your discussion is a bit hard to follow. I think you jump too much between reflecting noteworthy findings/expected versus unexpected results, the possible reasons why, and then broader discussion about the very interesting area.

I think think trying to reblock the discussion so that more concrete findings are presented first, and then later on more abstract thinking is considered, would significantly aid the reader.

You don't talk about your R-squared value in the discussion; I think it would be noteworthy to at least nod to the fact that this is an incredibly complex thing to model and there are lots of unmeasurable factors at play (such as those you describe in the discussion!)

Another thing perhaps to draw out even more, as you start to do, is the nuances re prescribing (SSRIs in adults versus children being at different thresholds, ADHD medications can be prescribed by GPs under shared care or by private/2ary care).

Page 7 Paragraph 3; I don't quite accept your point re talking therapy and cultural background. If they have already gone to their care provider re their MH, this does not explain why they wouldn't be afforded talking therapy?

Page 8 paragraph 2; note that they may not be starting stimulant medication, but rather may just be continuing stimulants from childhood?

Paragraph 3: "outside of the public health system"—ADHD is not a diagnosis made in the public health system—reword? Also—it is not really mentioned in your discussion that your ICS-wise approach does not consider the fact that the ICBs and MH trusts are at different levels of scale, and therefore more work trying to understand specifically how these smaller ICB units influence the wider MH trust (and whether any impact of affluence/socioeconomic factors) would be needed to support some of your reasoning here.

Interesting that you don't talk re ADHD in the last pg on page 6, where those issues re navigating healthcare may apply better in ADHD than to SSRIs (see MAP Study 2022-24).

May also be worth discussing the pandemic in your findings. Your range of years (2020-22) includes the tail end of the pandemic, where we know MH amongst CYPs was worse and the impact of pandemic measures was differential across sociodemographic gradients. This may play into the MH impact.

£51mn should be £51m

Implications for health policy;

This shouldn't be opening with a statement re wait times, when you have not looked at these. This section should open with something about what you found.

Limitations; reflect re IMD being an imperfect measure, nothing re waiting times in your data (which may indicate service stress/access problems), no measures of diagnoses made.

Conclusion:

I can't really see how you can justify saying that from your data, services are not well matched to needs based on your data. Either back this up a bit better in your discussion or remove.

**Reviewer**	**3**
**Name**	**Eberhard, Sophia**
**Affiliation**	**Lund University**
**Date**	**03-Feb-2025**
**COI**	**None**

This paper is of interest for both CAMH-professionals and researchers in the field. The paper highlights with robust data that inequalities occur both around diagnosing mental health conditions and treating them. This phenomenon has been noted previously all over Europe, but to my knowledge not analysed as thoroughly as in this paper. To readers from other countries, it would be of interest if the authors would elaborate more on how GPs act as gatekeepers, as this might differ within Europe. I would also suggesting adding more of information on how the rates of SSRI – and stimulant prescription in northern London correspond to other countries., and also how this corresponds to other regions in the UK, if such data is available.

VERSION 1 - AUTHOR RESPONSE

Reviewer: 1
Dr. Simone Amendola, Independent researcher
Comments to the Author:
Please refer to the attached file.

Dear Editor and Dear Authors,

I appreciate the invitation and opportunity to review the manuscript "Sociodemographic and geographical variation in prescribing psychotropic drugs to children and young people with common mental disorders and Attention Deficit Hyperactive Disorders in Northwest London: population-based study". The present analysis benefited from the use of a comprehensive coverage of SSRI and ADHD medications prescribed in the Northwest London region. It is indeed an interesting paper exploring associations between prescribing patterns and geographical indicators. According to my opinion, the clarity and rigor of the manuscript need to be further improved as well as the reporting in the results section and the discussion of some of the results. The use of a consistent structure when presenting the results and when discussing them (e.g. starting with presenting/discussing findings related to age first, then gender, the borough of residence, ethnicity, and lastly socioeconomic deprivation) may be of help in organizing the text and improving the reading flow. Below are my comments and suggestions. I would gladly review the second version of this manuscript. Best regards

RESPONSE: Thank you

Title and abstract

1.

Reading the abstract, it is evident that the analysis deals with SSRI and ADHD medications. I believe it would be better to specify this already in the title rather than using "psychotropic drugs for ... disorders". Or did the authors also analyze indications for prescribing those drugs (please note that this is not reported in the abstract)?

RESPONSE: We have revised the title to clarify what the study involved

2.

The time during which prescriptions were analyzed could be added to the abstract under "setting". 2020-2022 is reported among the results but it is unclear whether this was the period of interest for this study.

RESPONSE: We have now included the year range for the study in the abstract as it is the period of interest for the study

3.

Results are only partially reported and focus on deprivation in the current form. Preference needs to be given to results from logistic regression and divergent findings according to each drug group.

RESPONSE: We present results of both descriptive and regression analyses. We focus particularly on deprivation because this is a national and local priority. We do not agree that we should give preference to regression results. We have presented these results widely and there has consistently been a lot of interest in the descriptive analyses. We agree that there is a lot of interesting work that should follow, investigating other factors and using more sophisticated statistical methods but this beyond the scope of the current descriptive paper.

4.

Abstract conclusions: are you sure that they match your results? For example, it is stated that "Mental health care ... does not appear to be well matched to need and may be exacerbating inequalities.". However, did you analyze which type of intervention was suggested/provided besides drugs or CYP needs? This is not clear in the abstract. Furthermore, this is a cross-sectional study, and the data analyzed could not inform on cause-effect relationship (i.e. exacerbating inequalities). Under results, it was also reported that "sociodemographic factors could not explain most of the variation in prescription rates". Finally, what is intended with "More extensive use of linked electronic health records" may be also briefly clarified (e.g. with examples between brackets) in the abstract and addressed in the discussion section.

RESPONSE: This section has been revised to clarify these points

5.

May more specific analysis be needed to inform on the relationship between prescribing and socioeconomic deprivation, also considering the severity of mental health symptoms for which drugs were prescribed and other therapeutic interventions?

RESPONSE: We acknowledge that these descriptive analyses raise important questions about other treatments. However, the data available are limited. As more data fields are added to the North West London Discover dataset, we hope to be able to address treatment pathways in

more detail. We now highlight the potential for further analyses of treatment pathways in the Abstract and the Discussion.

Background

6.

The topic of pharmacological interventions for mental health difficulties is introduced on line 14. However, more contextual data is needed to provide a better picture for readers who are not from the UK. For example, are mental health difficulties of CYP primarily treated with drugs rather than other types of treatments? This section would benefit from a discussion of available evidence on preferred/recommended mental health treatments for CYP in the UK and London specifically, if any.

RESPONSE: Prescribing practice tends to follow NICE (National Institute for Health and Care Excellence) guidance. For common mental disorders (anxiety, depression) talking therapies are the usual first line intervention. In general, medication is used less in the UK than in the USA and is almost always prescribed by a specialist (e.g. a child psychiatrist or paediatrician) in children under the age of 16). This point has been mentioned in the Background in relation to the roles of primary care and specialists in the UK health system.

7.

It seems that this analysis was entirely exploratory despite focusing on sociodemographic factors (i.e. no specific hypothesis to be tested). This could be clarified at the end of the background section.

RESPONSE: We have revised the final paragraph of the background section to state that this is an explanatory study, with a particular focus on in geographical variation and deprivation (based on local and national NHS priorities).

Methods

8.

The reason to exclusively analyze SSRI and ADHD drugs among many other drugs available on the market needs to be clarified as well. Is this perhaps a limitation of the study that aimed to focus on psychotropic drugs for common mental disorders?

RESPONSE: We acknowledge that this is not a comprehensive list of all medicines used for mental health. We focussed on these medicines because: 1) they tend to be specific to mental health diagnoses; 2) they are well coded; 3) they are prescribed frequently enough to examine variation by borough and deprivation index, which would not apply to medications more rarely used in these populations such as antipsychotics and mood stabilisers. We have now included this in the Methods highlighting this rationale.

9.

Similarly, the reason to focus on the period 2020-2022 and not on the years before (from 2015 to 2020) needs some explanation. Such a choice might have also made possible the analysis of disparities in prescribing trends.

RESPONSE: These analyses aimed to describe current prescribing patterns in NW London in order to understand need and guide service development. We know that the prevalence and nature of MH problems in CYP has changed significantly during the COVID period, and so we did not include data from before 2020 as they do not reflect contemporary need.

10.

Could the focus on residents only represent a bias in this analysis? More than 150 thousand CYP were excluded. Did the authors test differences between included and excluded participants in the variables considered in this study? Any difference in prescription prevalence and sociodemographic indicators?

RESPONSE: The vast majority of those excluded were because they were not resident in NW London and so do not meet inclusion criteria for a population-based study. The approach adopted allows us to focus on the provision of treatment for those living in NWL.

11.

It would be good to add excluded drugs within each category, if any, to Appendix 1.

RESPONSE: As mentioned in point 9 (above), we selected medicines that are commonly prescribed (the range of medications in everyday use in the UK for mental disorders in children is limited).

Results

12.

The 95% CI for prevalence could be added to the column "yes" (i.e. prescription status) in Tables 1A and 1B.

RESPONSE: We do not consider the 95% CIs are necessary for these descriptives and think they would make the Table difficult to read.

13.

Table 2 is difficult to read in its current form. I would suggest excluding raw numbers to only report prevalence.

RESPONSE: We acknowledge that the Table [now Table 3] was difficult to read in manuscript form and have attempted to improve its clarity. Additionally, we have included a similar Table elsewhere and suggest that the Table will be clear when typeset for publication.

14.

To facilitate results comparison between bivariate association (table 1a and 1b) and multivariate association (table 3a and 3b) logistic regression models should be preferred to test bivariate associations (using the same reference category as in the multivariate models) and odds ratio could be added to table 1a and 1b rather than Chi-square test. The chi-square test alone says nothing about where the difference in the distribution occurred.

If the chi-square test is preferred standardized residuals should be reported and their effect interpreted.

RESPONSE: The main aim of this study is to describe variation in prescriptions, and the descriptive analyses has consistently received the most attention when these data are presented. We agree more detailed analyses would be interesting, but beyond the scope of this study.

15.

Considering that it is common to observe significant p-values with large samples, how did the authors discriminate whether their results were also (from a public health or clinical perspective) meaningful? Previous recommendations about OR effect size interpretation might help here. This would also guide the authors to focus on more important, key, or meaningful findings in their discussion. The following may be of help https://doi.org/10.1080/03610911003650383.

RESPONSE: We thank the reviewer for providing us with this reference. We acknowledge the reviewer's point that large sample sizes can lead to significant results when effect sizes are small. However, the effect sizes presented are large (3-4 fold differences between some groups). These differences are very significant from clinical and public health perspectives, and these findings have received a lot of attention from local commissioners and have informed plans for more equitable provision of care in NW London.

16.

I would encourage the authors to differentiate three sections of the results, a first one describing results from bivariate associations, a second one on those from multivariate analysis, and a third one describing the figures (subheading might be useful here).

RESPONSE: We acknowledge that the results were difficult to follow and have addressed this by restructuring this section, although we have not differentiated the figures from the analyses because they are not separate findings.

17.

Regarding the description of the figures, a clearer description could be that: in Figure 5a, the lower prescription of SSRI for all less deprived groups (compared to the most deprived, IMD1) is evident overall, except for Ealing and Hill. In Figure 5b there is more variability related to the findings from the logistic regression of ADHD drug prescription higher for the least deprived group (IMD5) compared to the reference (IMD1) (and no difference between IMD1 and other groups). Figure 5b suggests that the significant result is possibly primarily explained by prescriptions in Houns and HeF with Harrow representing an exception (where we see that the effect is reversed). However, all of this is a description and was not formally tested in a subgroup/moderation analysis. I believe that if the authors intend to focus on the discussion of these specific results they need to perform additional analysis. Lacking such an analysis, I recommend deleting the description from these results from the abstract.

RESPONSE: Although no formal statistical testing was carried out, we consider that the figures are useful for the reader. We have revised the description of the figures in the text to more clearly draw out the patterns observed. These figures have been the main focus of discussion when the findings have been presented and we consider that they are an important contribution to public debate about YP mental health in the UK.

Discussion

18.

Lines 11-12: There seems to be no linear relationship according to your findings but only that "the MOST deprived group" showed higher SSRI prescription compared to other less deprived and non-deprived groups. Please change accordingly.

RESPONSE: We accept that these are not linear relationships and have changed the text to reflect this.

19.

Lines 52: The authors found an effect of ethnicity even after controlling socioeconomic deprivation and geographic area of NWL and ascribed this effect to the possible occurrence of bias, stereotypes, and competency within the services. Is this difference between ethnicities observed in access to non-drug mental health interventions as well?

RESPONSE: Ethnic differences in accessing mental health care has been found in the nationally-representative Mental Health of Children and Young People in England Survey, with children from ethnic minorities less likely to contact any professional services (Mathews et la 2024; https://pubmed.ncbi.nlm.nih.gov/38172370/). Evidence from a scoping review of qualitative studies also demonstrates ethnic difference in care seeking and access to mental health care that arise from a range of factors, including lack of information and trust in professionals, and social stigma and cultural expections (Coelho et al, 2022; https://pubmed.ncbi.nlm.nih.gov/35944089/). We have included this is the Discussion.

20.

Page 7 on the bottom: "We found differences in ADHD prescription rates between those aged <18 years and those aged 18-24 years". I cannot find this analysis in the text. If you refer to the data displayed in Figure 1b, then the pattern is a bit more complex. There "seems" to be (because no test was conducted, maybe I am missing something here) a gradual linear increase in ADHD drug prescription in females while male prescription rates peaked at 18 years old and then decreased. These findings may merit further discussion.

RESPONSE: We have revised the Results section to more accurately reflect the patterns observed. Prescription rates by age are influenced by many interrelated factors, including healthcare seeking behaviour, provision of services, and evolving societal awareness/understanding of ADHD. It is beyond the scope of the paper to explore these factors in more detail.

21.

Line 25: "we found higher rates of ADHD prescriptions in less deprived areas". The difference was significant only between the least and the most deprived areas. The most deprived area did not differ from areas with "intermediate" deprivation. Please change accordingly.

RESPONSE: We have changed the text to clarify this point.

22.

Could the significant difference be because hyperactivity and inattention in the least deprived area may be considered socially inadmissible needing correction, something “out of order”? When this is not the case for the least deprived and intermediates areas? The authors could consider this additional explanation.

RESPONSE: We thank the reviewer for their interesting suggestion, which we are unable to investigate with the present data. However, we look forward to exploring this explanation in future work.

23.

A relevant part of the results section is focused on prescriptions according to London Borough. However, a discussion of the findings is completely lacking.

RESPONSE: Clinicians and many families in NW London are well aware of the different level of service provision in different areas (the “postcode lottery”) and have been very interested in seeing robust data that quantify the impact of these differences. We do not discuss these in detail as the mainly reflect differences in level of resources and historical factors about service provision. We have added a sentence to the manuscript on this.

24.

The discussion could be organized in subheadings, e.g. with one paragraph on social deprivation and another on geographical variation.

RESPONSE: We have edited and expanded the Discussion, which draws out variation by sociodemographic characteristics, including social deprivation and borough, but given that these factors are interrelated, we do not feel that it would be helpful to differentiate them using subheadings

25.

I also encourage the authors to assume a more critical perspective and include a paragraph discussing the limitations of this study.

RESPONSE: We have now included limitations in the Discussion.

26.

The section about study implications primarily relies on disparities in waiting times but this aspect was not analyzed in the present study. Please base the study implications on the study findings.

RESPONSE: We have now removed the discussion of waiting times.

27.

Conclusions need restructuring despite the fact that the first part (lines 17-22) is well integrated with the rest of the text. The authors could capitalize on that to highlight how their study informs or advances current knowledge, i.e. one or a few take-home messages resulting from the findings of the study.

RESPONSE: We have revised the Conclusions, stating that these findings may inform design and evaluation of local and national strategies to improve outcomes and reduce inequalities in young people's mental health.

Reviewer: 2
Dr. John Ward, University of Exeter
Comments to the Author:
Thank you for giving me the opportunity to review this interesting manuscript. I think that the subject matter, variations in prescribing considering social factors, is very important. I am impressed by the size of the dataset used in this study.

RESPONSE: Thank you

My main comment would be that the analysis is very interesting, but feels that the background/discussion and conclusion have been tacked on without a good narrative thread in the paper. A unifying thread through the paper would help make for a more robust argument.

RESPONSE: We thank the reviewer for their interest in the results. The rationale for the paper is to explore whether there was variation in prescribing by geographic and sociodemographic factors. While there may have been other factors at play, such as availability of mental health support, we were not able to study these and can only speculate what they might be and how they operate. This paper is part of a wider programme of work aiming to identify and overcome health inequalities in access to mental health care.

As a general comment, I think that Northwest should be two words (North West), but I will leave this as a journal house style matter.
RESPONSE: We have made this change

Specific comments;
Title: you are missing an "a" from "a population based study"
RESPONSE: The title has been revised to address editor and reviewer comments

Abstract:
Your results section needs tidying up; it reads as cumbersome and there are issues with the grammar.

RESPONSE: Thank you, we have revised the abstract.

Also, you say ADHD medication and ADHD drug; please be consistent.
RESPONSE: This has been changed to medication

Background:
I think that your background is far too short, and doesn't justify why you go onto the look at the question that you do.
I agree that CYP mental health is multi-faceted and prescribing is expensive, but I want to know

a bit more about why this justifies your study and how your study would help you address the problems you have identified. You sort of do this in the last paragraph of the background but it's quite vague and does not pin onto any existing literature. As another general note, you comment a couple of times own the costs of prescribing but for unclear reason (both in the background and in the discussion).
RESPONSE: We have restructured and added to the Background to give a better sense of the rationale for the study

Methods:
The description of the dataset is good; thank you.
Re the participants; you say that they have to be in the dataset in 2022, but the sample period is between 2020-22. Did they have to be in the dataset for this entire period, or just 2022?

RESPONSE: Participants were included in the dataset in 2022 if they were a child/young person in this year and had received a prescription in the years 2020-22 (regardless of whether they were included in the dataset for this entire period)

Was any de-duplication of the dataset required (I'm assuming not if by NHS number).

RESPONSE: Deduplication was not needed (pseudonymised NHS numbers are used as theidentifier in this dataset).

Was this study pre-registered (not that it had to be, but would be good to know for the reader).

RESPONSE: The study was registered with the Discover Data Access Group, who control access to the dataset. This is now mentioned in Ethics.

There is no justification as to why using prescription rates in this study? It would be good to know why specifically you have chosen prescribing patterns, and why specifically SSRIs and ADHD medications.

RESPONSE: We have addressed the query about the selection of medications in response to a point made by Reviewer 1.

Was the IMD rank for the GP practices or for the patients themselves?
RESPONSE: IMD was calculated at patient level using home postcode.

Results:
I would not use the word correlation unless you have calculated a correlation.

RESPONSE: We agree. We have rephrased those sentences as we have not calculated correlations.

“More deprivation in certain ones”—too informal.
RESPONSE: We have removed this text

Discussion:

Your discussion is a bit hard to follow. I think you jump too much between reflecting noteworthy findings/expected versus unexpected results, the possible reasons why, and then broader discussion about the very interesting area.

I think think trying to reblock the discussion so that more concrete findings are presented first, and then later on more abstract thinking is considered, would significantly aid the reader.

RESPONSE: We have revised the Discussion following helpful suggestions from the reviewers.

You don’t talk about your R-squared value in the discussion; I think it would be noteworthy to at least nod to the fact that this is an incredibly complex thing to model and there are lots of unmeasurable factors at play (such as those you describe in the discussion!)

RESPONSE: We have now mentioned this in the Discussion

Another thing perhaps to draw out even more, as you start to do, is the nuances re prescribing (SSRIs in adults versus children being at different thresholds, ADHD medications can be prescribed by GPs under shared care or by private/2ary care).

RESPONSE: Of note, these are unlikely to be prescriptions initiated in primary care; they are likely to have been initiated and monitored in secondary care by mental health professionals, with GPs providing subsequent repeat prescriptions.

Page 7 Paragraph 3; I don’t quite accept your point re talking therapy and cultural background. If they have already gone to their care provider re their MH, this does not explain why they wouldn’t be afforded talking therapy?

RESPONSE: As mentioned in response to a query by Reviewer 1, there is some evidence of ethnic differences in access to mental health services.

Page 8 paragraph 2; note that they may not be starting stimulant medication, but rather may just be continuing stimulants from childhood?
RESPONSE: This is true and we already show this indirectly in the age analyses.

Paragraph 3: “outside of the public health system”—ADHD is not a diagnosis made in the public health system—reword?

RESPONSE: We have clarified that this is the funded health system (NHS).

Also—it is not really mentioned in your discussion that your ICS-wise approach does not consider the fact that the ICBs and MH trusts are at different levels of scale, and therefore more work trying to understand specifically how these smaller ICB units influence the wider MH trust

(and whether any impact of affluence/socioeconomic factors) would be needed to support some of your reasoning here.

RESPONSE: These findings will have implications for both public health/commissioners and service-level planning.

Interesting that you don't talk re ADHD in the last pg on page 6, where those issues re navigating healthcare may apply better in ADHD than to SSRIs (see MAP Study 2022-24).

RESPONSE: We have now highlighted issues around navigating healthcare in the Introduction

May also be worth discussing the pandemic in your findings. Your range of years (2020-22) includes the tail end of the pandemic, where we know MH amongst CYPs was worse and the impact of pandemic measures was differential across sociodemographic gradients. This may play into the MH impact.
RESPONSE: We acknowledged that the prevalence and nature of MH problems in CYP changed significantly during the COVID period, and so we did not include data from before 2020 as they do not reflect contemporary need.

£51mn should be £51m
RESPONSE: We have corrected this

Implications for health policy;
This shouldn't be opening with a statement re wait times, when you have not looked at these. This section should open with something about what you found.

RESPONSE: We have removed the statement about waiting times.

Limitations; reflect re IMD being an imperfect measure, nothing re waiting times in your data (which may indicate service stress/access problems), no measures of diagnoses made.
RESPONSE: We thank the reviewer for these suggestions, which we have included in a section on Limitations in the Discussion section

Conclusion:
I can't really see how you can justify saying that from your data, services are not well matched to needs based on your data. Either back this up a bit better in your discussion or remove.
RESPONSE: We have removed this text

Reviewer: 3

Dr. Sophia Eberhard, Lund University
Comments to the Author:
This paper is of interest for both CAMH-professionals and researchers in the field. The paper highlights with robust data that inequalities occur both around diagnosing mental health conditions and treating them. This phenomenon has been noted previously all over Europe, but to my knowledge not analysed as thoroughly as in this paper. To readers from other countries, it would be of interest if the authors would elaborate more on how GPs act as gatekeepers, as this might differ within Europe. I would also suggesting adding more of information on how the rates

of SSRI – and stimulant prescription in northern London correspond to other countries., and also how this corresponds to other regions in the UK, if such data is available.

RESPONSE: Thank you. Primary care serves as a gatekeeping function in the United Kingdom, with General Practitioners (GPs) making referrals to specialist child and Adolescent Mental Health Services (CAMHS). Specialists usually undertake diagnosis and initiate medication, after which GPs are responsible for routine monitoring and repeat prescriptions under shared care arrangements (Newlove-Delgado et al, 2019; https://pubmed.ncbi.nlm.nih.gov/30730035/

VERSION 2 - REVIEW

**Reviewer**	**1**
**Name**	**Amendola, Simone**
**Affiliation**	**Independent researcher**
**Date**	**07-Jul-2025**
**COI**	

Dear Authors,

Thank you for considering all my comments to the previous version of this manuscript. I believe that its overall clarity improved in this new version. However, I still have two points which I think could be useful to improve this manuscript (see below).

To note, in your reply, you stressed (more than once) that you have presented the same "results widely and there has consistently been a lot of interest in the descriptive analyses". However, descriptive statistics are used and usually restricted to (national) reports rather than to journal scientific articles, which usually employ and benefit from more detailed analyses. For this reason, I suggest that the figures are described at the beginning of the results section or moved directly to a supplementary material file.

New comments:

1. In replying to my comment n.9 regaridng the time period of the data collection, you mentioned that the period before the COVID pandemic (from 2015 to 2020) was not analyzed because "we know that the prevalence and nature of MH problems in CYP has changed significantly during the COVID period, and so we did not include data from before 2020 as they do not reflect contemporary need.". However, does the period analysed (2020-2022) reflect "contemporary need" and "current prescribing patterns" in NW London? OR, might it only reflect "prescribing during COVID-19"? I think there may be a major bias for which the authors cannot conclude on whether they analysed "current prescribing" or, better, "prescribing under particular conditions" (COVID-19 pandemic). This should be considered when discussing the study limitations advocating for the analysis of more recent data "in order to understand need and guide service development.".

2. The discussion of the results related to the factor "London Borough" (LB) has not been improved as I suggested in my comment n.23. It is not clear why the authors did focus on variation in prescribing without trying to interpret the results they obtained. This is especially relevant to discuss as their findings are also somewhat contradictory: in predicting ADHD prescriptions all LB showed higher prescriptions compared to Ealing; whereas for SSRI findings were mixed with only two LB showing higher prescriptions compared to Ealing, three LB showed lower prescriptions compared to Ealing, and two LB showed no difference compared to Ealing. The authors need to provide potential interpretations for their findings. It is not clear whether the authors noticed that.

**Reviewer**	**2**
**Name**	**Ward, John**
**Affiliation**	**University of Exeter**
**Date**	**07-Jul-2025**
**COI**	

Thank you for taking the time to make the changes suggested in the previous round of revisions, I have only a few minor comments further to what I have said previously.

Overall, the paper reads a lot better and there is a clearer narrative thread. There are a few typos in places (“anSSRI”, “child and Adolescent”, “most treated with SSRI”, and unclosed []), which means another good proof read is required.

Regarding the background, more clearly stating that you are interested in variation would be good earlier on, as it would frame the background better for the reader. I would also suggest not saying that the pathway is likely to be complex, as this makes it sound as if you are unsure (when there is lots of evidence that it is, and lots of it you have cited already!).

Thank you for making my other recommended changes.

VERSION 2 - AUTHOR RESPONSE

**Reviewer 3**:<br>I would also suggesting adding more of information on how the rates of SSRI – and stimulant prescription in northern London correspond to other countries., and also how this corresponds to other regions in the UK, if such data is available.	*We thank the reviewer and editors for this suggestion. We have added material on UK and international trends in prescribing of SSRI and ADHD medication for CYP in the discussion.*
**Reviewer 2:**<br>Thank you for taking the time to make the changes suggested in the previous round of revisions, I have only a few minor comments further to what I have said previously. Overall,	*Thank you*

the paper reads a lot better and there is a clearer narrative thread.	
There are a few typos in places (“anSSRI”, “child and Adolescent”, “most treated with SSRI”, and unclosed []), which means another good proof read is required.	*We apologise for the typos; all authors have contributed to further proof reading and corrected these errors*
Regarding the background, more clearly stating that you are interested in variation would be good earlier on, as it would frame the background better for the reader. I would also suggest not saying that the pathway is likely to be complex, as this makes it sound as if you are unsure (when there is lots of evidence that it is, and lots of it you have cited already!).	*We now state early in the introduction that our interest is in variation in prescribing. We have also rewritten the sentence on the patient journey, removing the reference to it likely being complex.*
Reviewer 1, Dr. Simone Amendola, Independent researcher<br><br>Thank you for considering all my comments to the previous version of this manuscript. I believe that its overall clarity improved in this new version.<br><br>However, I still have two points which I think could be useful to improve this manuscript (see below).<br><br>To note, in your reply, you stressed (more than once) that you have presented the same "results widely and there has consistently been a lot of interest in the descriptive analyses". However, descriptive statistics are used and usually restricted to (national) reports rather than to journal scientific articles, which usually employ and benefit from more detailed analyses.<br><br>For this reason, I suggest that the figures are described at the beginning of the results section or moved directly to a supplementary material file.	*Thank you for this comment.*<br><br>*We agree with the suggestion about the figures included and now take a more selective approach to presentation of the results, moving most of the figures to a supplementary file. We are happy to take editorial guidance on further exclusion of figures from the main paper.*
**Additional comments**	
1. In replying to my comment n.9 regaridng the time period of the data collection, you mentioned that the period before the COVID pandemic (from 2015 to 2020) was not analyzed because "we know that the prevalence and nature of MH problems in CYP has changed significantly during the	*This point is well taken – we agree that there are three phases: ‘pre covid, during covid, post covid’. From other public health pandemics we have learnt that the overall levels of mental health in the population deteriorates and then takes some time to readjust. We are still in that adjustment*

COVID period, and so we did not include data from before 2020 as they do not reflect contemporary need.".<br><br>However, does the period analysed (2020-2022) reflect "contemporary need" and "current prescribing patterns" in NW London? OR, might it only reflect "prescribing during COVID-19"?<br><br>I think there may be a major bias for which the authors cannot conclude on whether they analysed "current prescribing" or, better, "prescribing under particular conditions" (COVID-19 pandemic).<br><br>This should be considered when discussing the study limitations advocating for the analysis of more recent data "in order to understand need and guide service development.".	*phase and this may be reflected in prescribing rates, which may underestimate of the size of the problem. We have added a comment in the limitations to the effect that the data reflect prescribing within the context of the pandemic, and that ongoing monitoring of prescribing practices is needed in order to understand need and guide service development.*
2. The discussion of the results related to the factor "London Borough" (LB) has not been improved as I suggested in my comment n.23. It is not clear why the authors did focus on variation in prescribing without trying to interpret the results they obtained. This is especially relevant to discuss as their findings are also somewhat contradictory: in predicting ADHD prescriptions all LB showed higher prescriptions compared to Ealing; whereas for SSRI findings were mixed with only two LB showing higher prescriptions compared to Ealing, three LB showed lower prescriptions compared to Ealing, and two LB showed no difference compared to Ealing. The authors need to provide potential interpretations for their findings. It is not clear whether the authors noticed that.	*We acknowledge that there is value in highlighting differences in prescribing patterns between boroughs, including possible mechanisms. We have added more material on borough differences in the revised paper, results and discussion.*

VERSION 3 - REVIEW

Reviewer **1**

Name **Amendola, Simone**

Affiliation **Independent researcher**

Date **19-Sep-2025**

COI

Dear Authors,

I would like to express my appreciation for your thoughtful approach in revising the manuscript. The new version is clearer and more balanced in recognizing the study strengths and limitations. Therefore, I am pleased to recommend this article for publication in BMJ Open even though minor revisions are necessary, in my opinion, which could be made at the proof stage, but I defer to the Editor's decision on this.

I agree with the authors on the importance of acknowledging that sociodemographic factors alone did not explain most of the variation in prescription rates in this study. Individual factors (e.g., diagnosis of mental disorders, symptom severity, substance use) and socio-relational factors (e.g., family conflict, peer support, substance use) may contribute to this variation. Additionally, health-related economic factors, such as non-drug public spending and the prescribing attitudes of mental health professionals, may also play a role in explaining differences in prescription rates and it might have been good to mention them when discussing the study limitations. I have previously explored this topic in an analysis conducted in Italy (doi: 10.1097/YIC.0000000000000522; but please note that I do NOT suggest citing this reference as part of the review process). I believe it would be valuable for future research to investigate the potential impact of these factors on prescription rates.

As a final note, I recommend caution and would remove the sentence added to the discussion because to me it seems a "stretching" in interpreting the results (lines 40-45, p.12 highlighted version; "[…] findings such as particularly high ADHD prescription rates for those living in the least deprived areas in some boroughs compared to others suggests the need to improve access in areas with low rates of prescription and a focus on equity, including access to private diagnostic services and incentives for ADHD prescriptions."). The prescription rate is not a very good indicator of mental healthcare access or "equity", especially considering that data about other interventions were not analyzed in this study.

I wish the authors the best of success with their future endeavors.

Kind regards,

SA

VERSION 3 - AUTHOR RESPONSE

Comments to author	Response
Reviewer 1, Dr. Simone Amendola, Independent researcher<br><br>I would like to express my appreciation for your thoughtful approach in revising the manuscript. The new version is clearer and	*We thank the reviewer for their positive response to the most recent revision of this paper.*

more balanced in recognizing the study strengths and limitations. Therefore, I am pleased to recommend this article for publication in BMJ Open even though minor revisions are necessary, in my opinion, which could be made at the proof stage, but I defer to the Editor's decision on this.	
I agree with the authors on the importance of acknowledging that sociodemographic factors alone did not explain most of the variation in prescription rates in this study. Individual factors (e.g., diagnosis of mental disorders, symptom severity, substance use) and socio-relational factors (e.g., family conflict, peer support, substance use) may contribute to this variation. Additionally, health-related economic factors, such as non-drug public spending and the prescribing attitudes of mental health professionals, may also play a role in explaining differences in prescription rates and it might have been good to mention them when discussing the study limitations. I have previously explored this topic in an analysis conducted in Italy (doi: 10.1097/YIC.0000000000000522; but please note that I do NOT suggest citing this reference as part of the review process). I believe it would be valuable for future research to investigate the potential impact of these factors on prescription rates.	*We accept this important point which we acknowledge in the Discussion as a limitation, We now highlight other factors that might be implicated.*
As a final note, I recommend caution and would remove the sentence added to the discussion because to me it seems a "stretching" in interpreting the results (lines 40-45, p.12 highlighted version; "[...] findings such as particularly high ADHD prescription rates for those living in the least deprived areas in some boroughs compared to others suggests the need to improve access in areas with low rates of prescription and a focus on equity, including access to private diagnostic services and incentives for ADHD prescriptions."). The prescription rate is not a very good indicator of mental healthcare access or "equity", especially considering that data about other interventions were not analyzed in this study.	*We accept this point and have removed the text identified by the reviewer.*
